# High validity of measuring the width and volume of medial meniscal extrusion three-dimensionally using an MRI-derived tibial model

**DOI:** 10.1186/s40634-019-0216-2

**Published:** 2020-01-03

**Authors:** Goro Watanabe, Kenji Hoshi, Yasuo Kurose, Kazuyoshi Gamada

**Affiliations:** 1 Graduate School of Medical Technology and Health Welfare Sciences, Hiroshima International University, 555–36 Kurosegakuendai, Higashihiroshima City, Hiroshima, Japan; 2 Department of Orthopaedics, Hiroshima Prefectural Rehabilitation Center, 295-3 Taguchi Saijo-cho, Higashihiroshima City, Hiroshima, Japan

**Keywords:** Medial meniscus, Three-dimensional models, CT-derived models, Tibial plateau, Osteophyte

## Abstract

**Background:**

Medial meniscal extrusion (MME) is an important marker of knee osteoarthritis (KOA) progression. The purposes of this study were: 1) to determine whether there are morphological differences between CT- and MRI-derived tibial plateau models; and 2) to determine whether measurement of MME volume and width using an MRI-derived tibial model is as accurate as measurements on a CT-derived tibial model.

**Methods:**

This was a cross-sectional study that enrolled ten participants with medial KOA (Kellgren-Lawrence grade 1 to 3). Primary outcome was surface difference of the medial tibial plateau between CT- and MRI-derived models. Furthermore, volume and cross-sectional area of the medial tibial plateau were compared between CT- and MRI-derived models. Measurements of MME volume and width were compared between CT- and MRI-derived tibial models.

**Results:**

Minimal and maximal surface differences of the medial tibial plateau between the CT- and MRI-derived models were − 0.15 [− 0.44, 0.14] mm (mean [95% confidence interval]) and 0.24 [− 0.09, 0.57] mm, respectively. There were no significant differences in volume and cross-sectional area of the medial tibial plateau between CT- and MRI-derived tibial models. The MME volumes measured on CT- and MRI-derived models were 942.6 [597.7, 1287.6] mm^3^ and 916.2 [557.9, 1274.6] mm^3^, respectively (*p* = 0.938). The MME widths measured on CT- and MRI-derived models were 4.2 [1.9, 6.5] mm and 4.5 [2.2, 6.9] mm, respectively (*p* = 0.967).

**Conclusions:**

CT- and MRI-derived models of the medial tibial plateau did not show significant morphological differences. Both CT- and MRI-derived tibia can be used as a reference to measure MME in early-to-moderate medial KOA.

## Introduction

Medial meniscal extrusion (MME), with or without meniscal injury, is an important marker of knee osteoarthritis (KOA) progression. Both Kawaguchi et al. [1] and Yanagisawa et al. [2] demonstrated that MME width was greater in patients with higher Kellgren-Lawrence (KL) grades. In addition, the Multicenter Osteoarthritis (MOST) study found that the presence of MME predicted cartilage loss after thirty months with odds ratios of 2.02 and 3.62 for slow cartilage loss and fast cartilage loss, respectively [3]. Accurate MME measurements would be important for evaluating the efficacy of interventions as well as KOA progression [4]. Although many studies support the association between KOA progression and MME width, the method of MME measurement utilized only a two-dimensional (2D) MRI slice, which might not have measured the greatest extrusion width. Furthermore, validation studies for this method are lacking.

Our ability to assess the MME and osteophytes around the tibial plateau three-dimensionally may be limited. To improve longitudinal assessments of changes in MME volume and width, effects of the osteophytes should be eliminated. Therefore, Computed tomography (CT) could represent a valid alternative since CT demonstrates sharp bone contours and allows automatic or semi-automatic segmentation in a reproducible manner [5, 6]. In addition, CT had higher ability to detect bony erosions than MRI [7]. For example, two previous studies concluded that both CT-derived bone model and MRI-derived model provide highly accurate data [8, 9]. On the other hand, White et al. [10] used a digital caliper to determine that the CT-derived model was 0.9% larger and the MRI-derived model was 3.5% smaller than direct measurements of the femur and tibia, concluding that the MRI-derived model would not offer a feasible alternative to the CT-derived model due to size inaccuracies [10]. Moreover, detecting the contours of osteophytes around the tibial plateau yields greater error on MRI because these structures demonstrate a gradual histological appearance on MRI [11]. Therefore, questions remain as to whether the MRI-derived model provides accurate contour information in KOA and whether conventional 2D measurements of MME width is valid and reliable as compared with three-dimensional (3D) measurement.

In fact, the contour of the tibial plateau serves as a reference point for measuring MME. Several studies chose the outermost margin of the tibial plateau as the reference after researchers manually excluded any osteophytes [4, 12, 13]. However, this method may involve measurement bias due the subjective judgments involved in excluding osteophytes. 2D measurements of MME largely depend on whether the particular MRI slice used for the measurement reflects the true maximal MME width, while 3D measurement of MME using common coordinate systems allows researchers to obtain true volume data that is not biased by slice selection and/or knee position during scanning.

This study aimed to determine: 1) whether there are morphological differences between CT- and MRI-derived tibial plateau models, 2) whether measuring MME volume and width on MRI-derived tibial models is as accurate as measurements using CT-derived tibial models. The hypotheses of this study were: 1) volume and area of the CT-derived tibial plateau would be larger than those derived from MRI; and 2) MME width measured on 2D MRI slices is inaccurate compared with 3D measurement on CT-derived tibial models combined with MRI-derived meniscal models.

## Methods

This was a cross-sectional study based on image data obtained as baseline data in a randomized controlled trial (RCT) investigating the effect of exercise therapy in patients with KOA. This RCT was approved by the institutional review board of Hiroshima Prefectural Rehabilitation Center and Hiroshima International University (approval number: 19–029). Participants of this study were recruited from patients consulting the Department of Orthopaedics at Hiroshima Prefectural Rehabilitation Center. Informed consent was obtained from each of the individual participant included in this study in accordance with the Helsinki Declaration.

Inclusion criteria were Japanese aged from fifty to eighty years old, primary KOA, and KL grade from 1 to 3. Exclusion criteria were valgus KOA, secondary KOA, a history of knee surgery or knee injury, a history of other somatic diseases that could pose a risk, mental disorders, and communication difficulty. 327 patients were assessed for eligibility and 39 patients met the criteria. Ten subjects in this study were selected based on the additional procedure to choose three or four patients randomly from the stratified pools of patients based on the KL grade 1–3.

CT images were obtained using a clinical X-ray CT scanner (Aquilion TSX-101A, Toshiba Medical Systems, Ohtawara, Japan). CT scan was taken using axial slices, kilovoltage: 120 kVp, tube current: 70 mA, exposure time: 500 ms, exposure: 70 mAs, slice thickness: 0.50 mm, sampling 150 × 150 mm in-plane sampling, and imaging matrix: 512 × 512. MRI images were obtained using a 1.5 Tesla MRI (MAGNETOM Aera, Siemens, Munich, Germany), and knee coil (Tx/Rx 15-Channel Knee Coil, Siemens, Munich, Germany). MRI were obtained as coronal slices of proton density sequences, slice thickness: 2.0 mm, intersection gap: 0 mm, slice resolution: 180 × 180 mm, imaging matrix: 384 × 384, TE time: 11 ms, TR time: 3810 ms. Participants were in the supine position with the foot elevated so that the examined knee was fully extended.

Manual segmentation was utilized to differentiate osteophytes and the other tissues using 3D modeling software (3D-DOCTOR, Able Software Corp. Lexington, MA). Geometric bone models and medial meniscus (MM) models were created by segmenting the exterior cortical bone edges and MM edges. Created 3D models were converted to polygonal surface models. Smoothing was applied using a reverse engineering software (Geomagic Studio, Geomagic Inc., Research Triangle Park, NC).

**Fig. 1.**
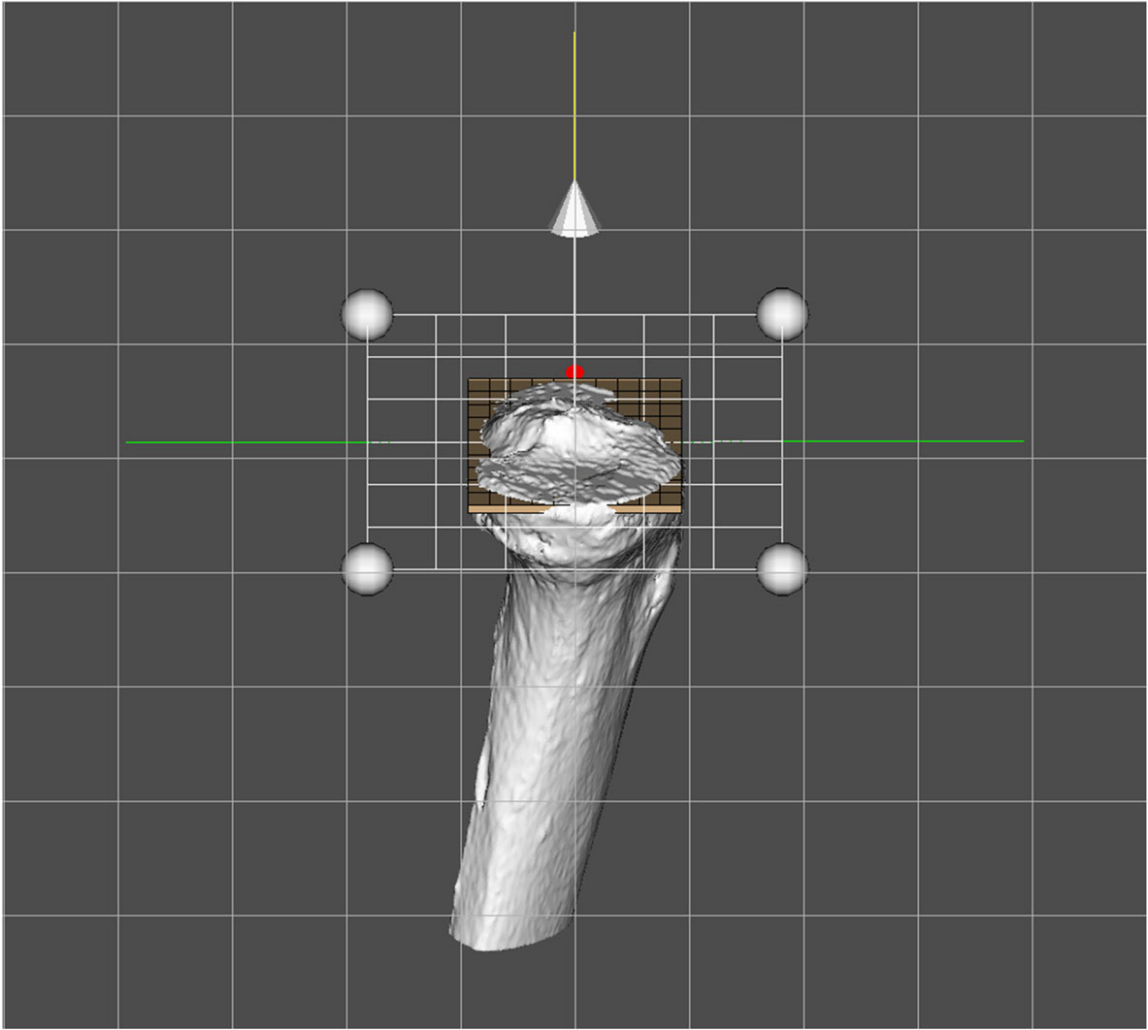
A local coordinate system of the tibia using the CT-derived tibial model. Tibial coordinate system is embedded using a virtual rectangle fitted onto the tibial cross section at the top of the fibular notch level. Then, the fitted rectangle is translated superiorly to the bottom of the medial and lateral tibial plateaus

A tibial coordinate system was embedded on the CT-derived tibia, which then was best-fitted to the MRI-derived tibia in order to share a common local coordinate system. A single experienced researcher embedded the local tibial coordinate system onto the CT-derived tibial models using commercial software (3D-Aligner, GLAB Corp., Higashihiroshima, Japan). On the CT-derived model, a virtual rectangle parallel to the tibial plateau plane was fitted onto the tibial plateau contours at the top of the fibular notch of the tibia in order to avoid osteophytes on the osteoarthritic tibial plateau [14]. Four sides of the rectangle were fitted onto the tangent of the posterior contours of the medial and lateral tibial condyles, the medial and lateral tangents of the medial and lateral tibial condyles, and the anterior tangent of the medial tibial condyle [14]. Then, the fitted rectangle was transferred superiorly to the bottom of the medial tibial plateau (Fig. 1.). The origin of the tibial local coordinate system was defined as the center of the rectangle, and medial/lateral (Z) axes and anteroposterior (X) axes were defined as two axes of the rectangle. The superior/inferior (Y) axis was defined as the cross product of the X- and Z-axes. Intra-researcher errors of the OA tibia on the X-, Y-, and Z-axis were (translation/rotation) 0.56 [0.22, 0.91] mm/0.86 [0.32, 1.40]°, 0.15 [0.08, 0.23] mm/0.39 [0.28, 0.50]°, and 0.21 [0.03, 0.40] mm/0.78 [0.28, 1.28]°, respectively [15]. The local tibial coordinate system of the MRI-derived model was embedded using the iterative closest point algorithm in Geomagic Studio.

**Fig. 2:**
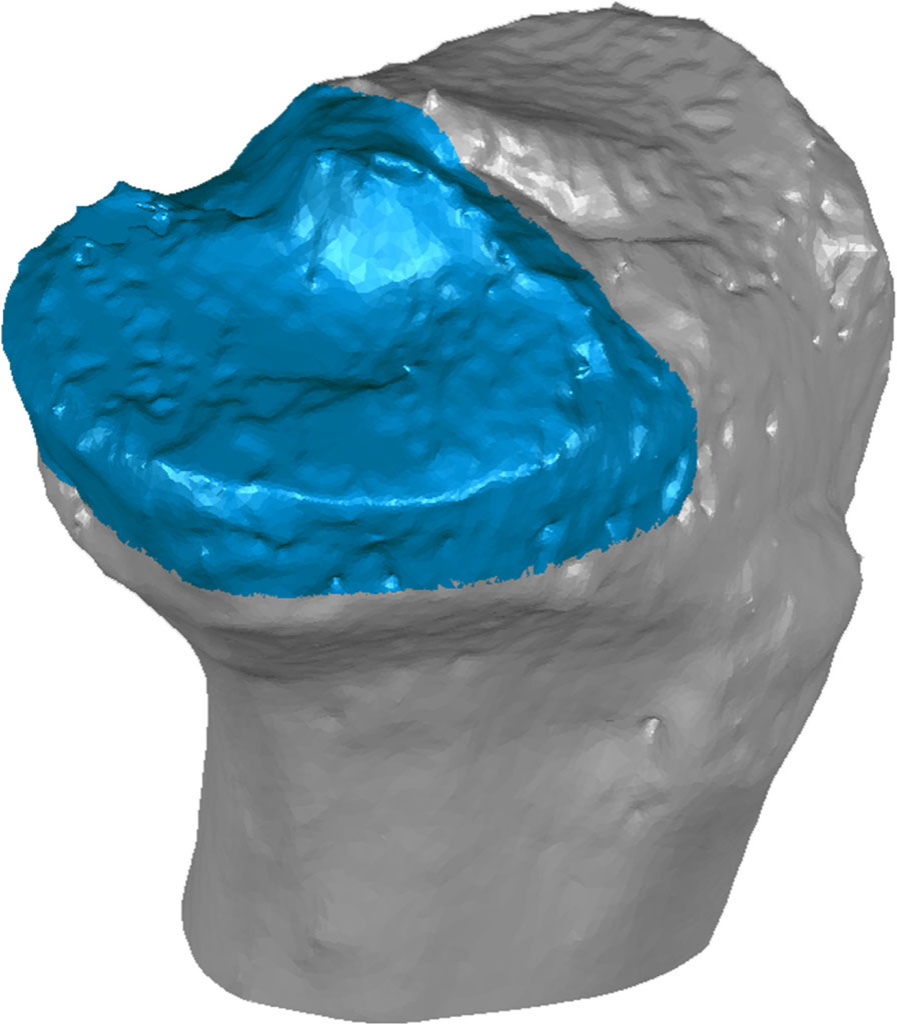
Model of the medial tibial plateau. The medial plateau model (blue) and the tibial model (gray) is shown. The medial tibial model is created by cutting the original tibial bone model at the level of 10.0 mm inferior to the tibial plateau (the ZX plane) and the original XY plane

**Fig. 3:**
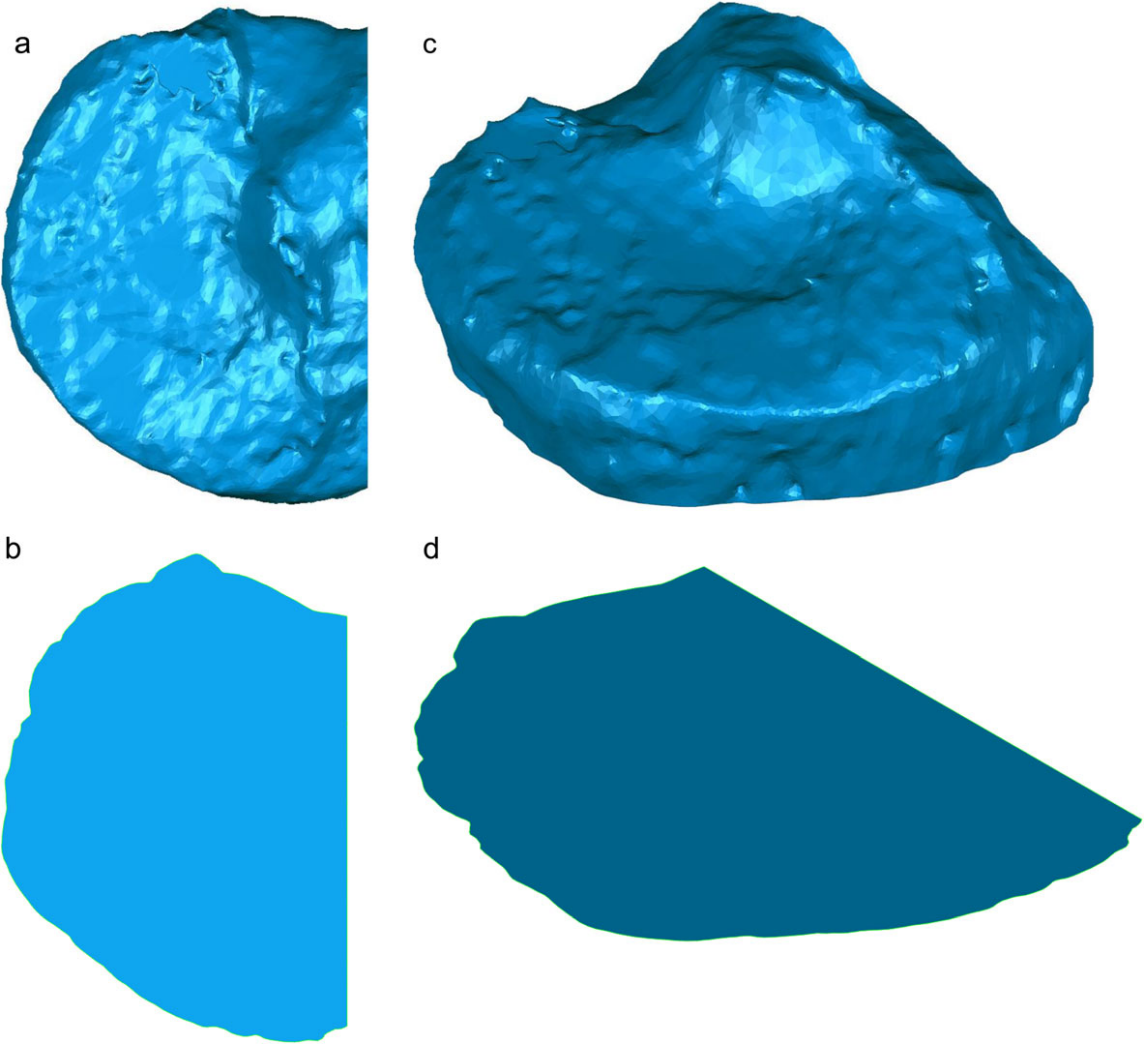
Cross-sectional model of the medial tibial plateau. Cross-sectional model is a cross section of the medial tibial plateau model at the level of 10.0 mm inferior to the origin parallel to the ZX plane. **a** Axial view of the medial tibial plateau model. **b** Axial view of the cross-sectional model of the medial tibial plateau. **c** Anteromedial view of the medial tibial plateau model. **d** Anteromedial view of the cross-sectional model of the medial tibial plateau

**Fig. 4:**
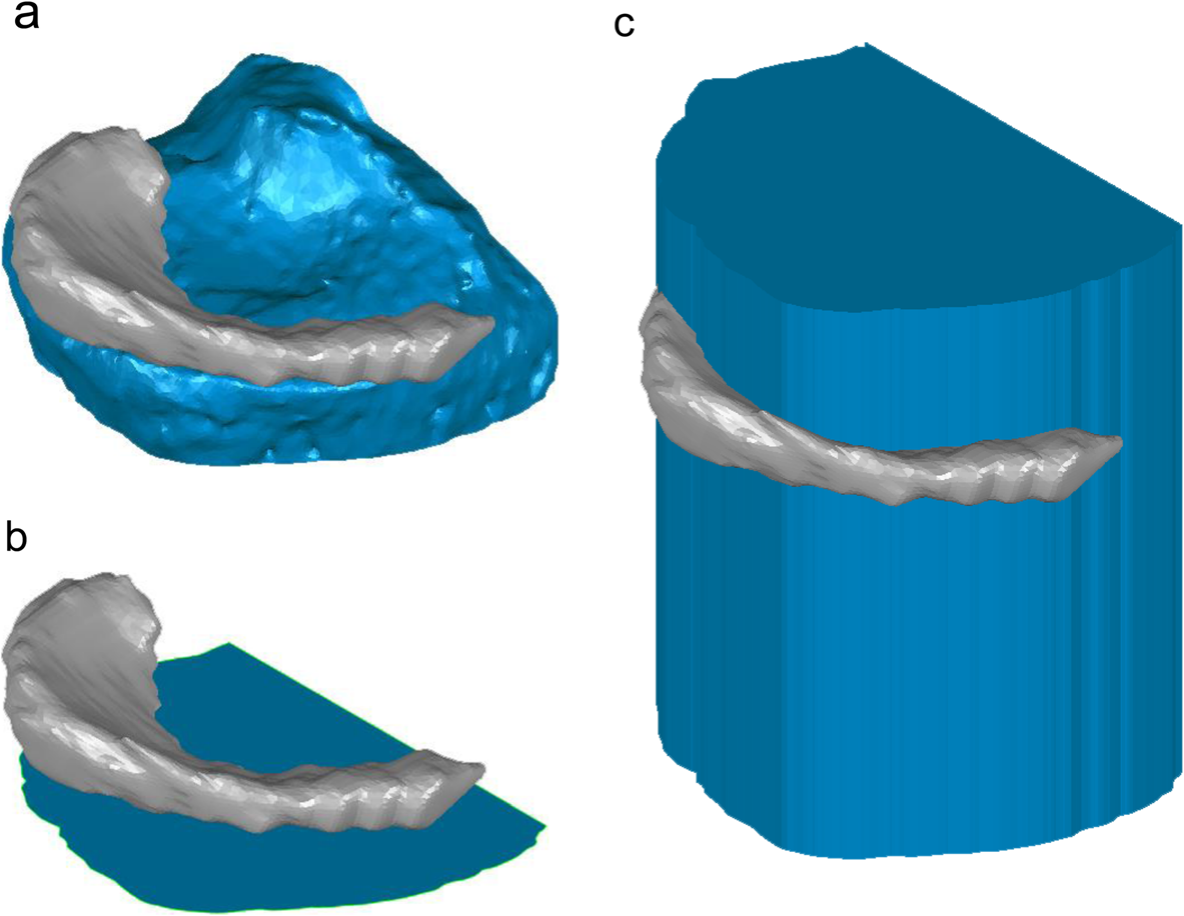
Volume of the medial meniscal extrusion (MME) model. The medial meniscus (MM) is shown in gray color. **a** The MM (gray) and the medial tibial plateau model (blue). **b** The MM (gray) and the cross-sectional model of the medial tibial plateau (blue). **c** MME model (gray) and the thickened cross-sectional model. The MME volume is defined as the volume of the MM model outside the thickened cross-sectional model

**Fig. 5:**
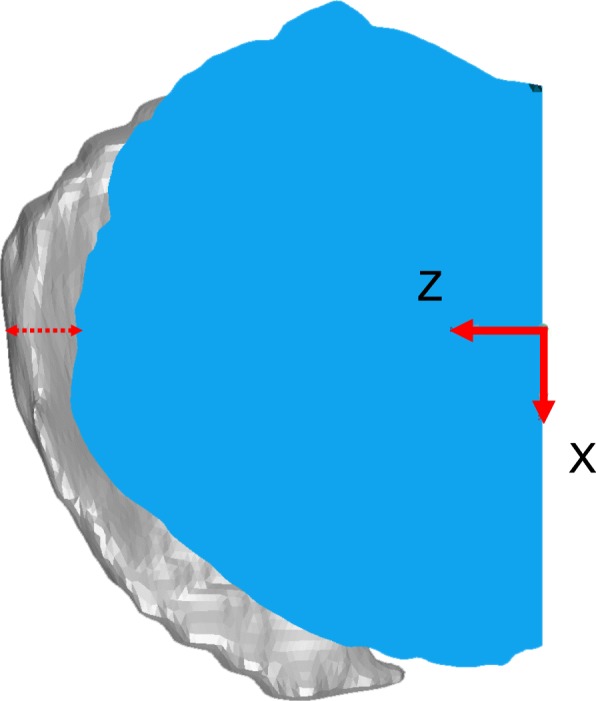
Width of the medial meniscal extrusion (MME) model. The MME model (gray) and the thickened cross-sectional model are shown from the axial view. The MME width is the distance from the outer edge of the thickened model to the outer edge of the medial meniscus model through the Z-axis

Surface differences and volume of the medial tibial plateau model were calculated using Geomagic Studio. A model of the medial tibial plateau was created by cutting the original tibial bone model at the level of 10.0 mm inferior to the origin parallel to the ZX plane and at the original XY plane (Fig. 2). The osteophytes were visually observed, and the obvious protrusion of bone were recognized as an osteophyte. The contour of the plane 10.0 mm below the tibial plateau plane was observed and bony protrusion was recognized as osteophyte at the observed plane. Cross-sectional model was a cross section of the medial tibial plateau model at the level of 10.0 mm inferior to the origin parallel to the ZX plane (Fig. 3). Then, the contour of the cross-sectional model was thickened superiorly for 30.0 mm to create a thickened cross-sectional model. MME volume was defined as the volume of the MM model outside the thickened cross-sectional model (Fig. 4). MME width was the distance of MME outside the thickened model on the Z-axis (Fig. 5).

**Fig. 6:**
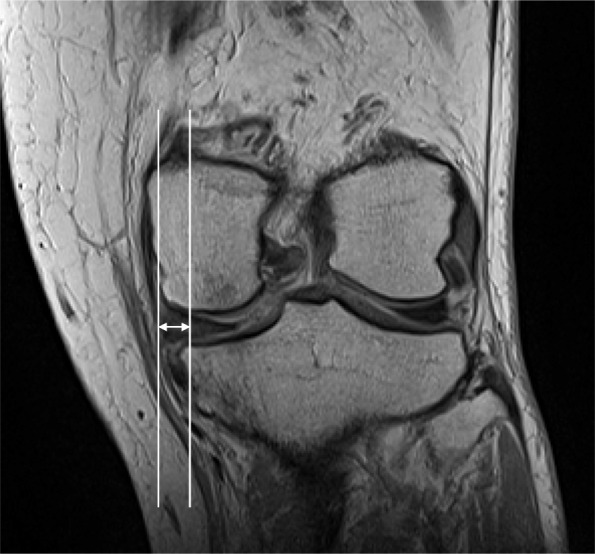
Measurement of two-dimensional (2D) medial meniscal extrusion (MME) width. 2D MME width is defined as the distance from the most extruded edge of the medial meniscus to the edge of the medial tibial plateau. The reference point for 2D MME width is the tibial plateau without the osteophyte. A vertical line is drawn connecting the femur and the reference point

2D MME width was measured using image processing software (ImageJ 1.50i, Wayne Rasband, USA). 2D MME width was defined as the distance from the most extruded edge of the MM to the edge of the medial tibial plateau [16]. The coronal slice showing the greatest area of the medial tibial spine was selected. The tibial reference point for 2D MME width was the edge of the bony contour of the tibial plateau without an osteophyte. A vertical line connecting the femur and the reference point was drawn (Fig. 6). A single observer performed measurements of the 2D MME width, MME volume, and MME width to assess intra-researcher reproducibility using interclass correlation coefficient (ICC). These measurements were conducted twice for eight subjects at an interval of one week.

### Statistical analysis

Sample size of this study was calculated using a power analysis program (G*Power version 3.1.9.2, Franz Faul, Germany). A power analysis was conducted using the result of the previous study [17]. 0.82 ± 0.62 mm were the surface difference between the CT- and MRI-derived tibial bone models. Effect size was 1.32. Calculations using an effect size of 1.32, α = 0.05, and power 0.95 showed that the required sample size was ten.

All variables were tested with Shapiro-Wilk test in order to determine the normality. Mean and 95% confidence interval were used to assess the demographic characteristics. Two-sample t-test was used to test each variable of the CT- and MRI-derived models. Linear regression analysis was carried out in order to test consistency of the medial tibial plateau volume and the cross-sectional area between the CT-derived models and MRI-derived models. Next, linear regression analysis was carried out to test consistency of the MME volume and width between the CT-derived models and MRI-derived models. In both analyses, data from the CT-derived model were dependent variables and data from the MRI-derived model were independent variables. Lastly, linear regression analysis was performed in order to assess the consistency between the 3D MME width and the 2D MME width obtained from an MRI slice. Statistical analysis was performed using commercial software (SPSS Statistics version 21, IBM Corp., Armonk, NY). The level of significance was set at α = 0.05.

## Results

Age, height, body mass, and body mass index (BMI) of the participants were 69.5 [65.5, 73.5] (mean [95% confidence interval]) years, 157.8 [153.6, 162.1] cm, 61.9 [53.1, 70.8] kg, and 25.1 [20.9, 29.2] kg/m^2^, respectively (Table 1). Three males and seven females were included; there were three, three, and four participants in KL grades 1, 2 and 3, respectively.

**Table 1:** Characteristics of the participants

Variable			*P*-value
Age (years-old)	69.5	[65.5, 73.5]	0.210
Height (cm)	157.8	[153.6, 162.1]	0.090
Weight (kg)	61.9	[53.1, 70.8]	0.086
Body Mass Index (kg/m^2^)	25.1	[20.9, 29.2]	0.075
Kellgren-Lawrence grade 1/2/3	3/3/4		

All variable except Kellgren-Lawrence grade are shown as mean [95% confidence interval]. *P* value are calculated using Shapiro–Wilk test.

ICC for 2D MME width for intra-researcher agreement was 0.990 [0.971, 0.997]. ICC for measuring the MME volume was 0.998 [0.992, 1.000]. ICC for measuring the MME width was 0.983 [0.924, 0.996].

All the participants had osteophyte in the medial tibial plateau, and none of ten had small osteophytes at 10.0 mm below the tibial plateau plane. All the participants except one could fully extend their knees. One participant with KL grade 3 had flexion angle of 4.5° by calculating with 3D bone models during MRI acquisition. MME volume and MME width using the CT- and MRI-derived tibia, and cross-sectional area of the medial tibial plateau using the CT- and MRI-derived tibia were normally distributed (Table 2). However, volume of the medial tibial plateau using the CT- and MRI-derived tibia were not normally distributed (Table 2).

**Table 2:** Demographic of volume and cross-sectional area of the medial tibial plateau models and volume and width of the medial meniscal extrusion

Variable		CT-derived model			MRI-derived model			*P* value	Effect size	Power
		Representative value		*P* value	Representative value		*P* value			
Medial tibial plateau	Volume (mm^3^)	13,031	(5300)	0.013	12,628	(4373)	0.019	0.912	0.08	0.91
	Cross-sectional area (mm^2^)	1484	[1338, 1630]	0.211	1,493	[1340, 1646]	0.122	0.906	0.04	0.91
Medial meniscal extrusion	Volume (mm^3^)	942.6	[597.7, 1287.6]	0.540	916.2	[557.9, 1274.6]	0.569	0.938	0.05	0.91
	Width (mm)	4.2	[1.9, 6.5]	0.242	4.5	[2.2, 6.9]	0.155	0.967	0.12	0.80

Volume of the medial tibial plateau is shown as median (inter quartile range), and the other are shown as means [95% confidence interval]. *P*-values of CT- and MRI-delived model are based on the Shapiro-Wilk test

**Fig. 7:**
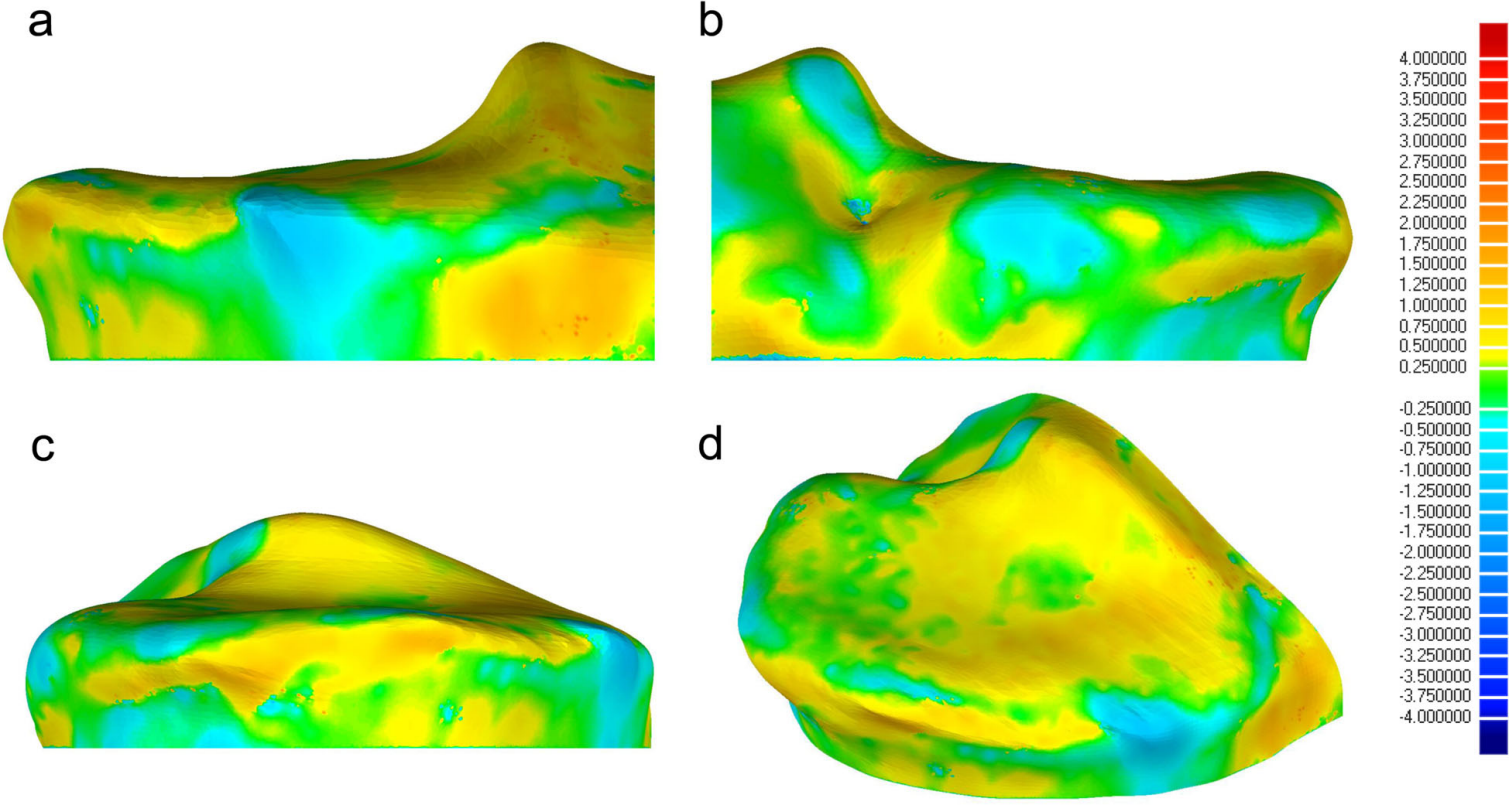
Representative case showing surface differences on medial tibial plateau models. **a** anterior view. **b**. Posterior view. **c** Medial view. **d** View from the upwards

Minimal and maximal surface differences of the medial tibial plateau between the CT- and MRI-derived models were − 0.13 [− 0.42, 0.16] mm (mean [95% confidence interval]) and 0.23 [− 0.10, 0.56] mm, respectively (Table 3, Fig. 7). The volume of the medial tibial plateau from the CT- and MRI-derived models were 13,031 (5300) mm^3^ (median (inter-quartile range)) and 12,628 (4373) mm^3^ (*p* = 0.912, d = 0.08, power = 0.91), respectively (Table 2). The cross-sectional area of the medial tibial plateau from the CT- and MRI-derived models was 1484 [1338, 1630] mm^3^ (mean [95% confident interval]) and 1493 [1340, 1646] mm^3^ (*p* = 0.906, d = 0.04, power = 0.91), respectively. Therefore, there were no significant differences in the volume and cross-sectional area of the medial tibial plateau between the MRI-derived and CT-derived tibial models. Linear regression analysis demonstrated that the volume of the medial tibial plateau of the CT- and MRI-derived models showed a high consistency (F(1, 9) = 8015, *p* < 0.001) with adjusted R^2^ of 0.999 (Table 4). Linear regression analysis also demonstrated that the cross-sectional area of the medial tibial plateau of the CT- and MRI-derived models showed a high consistency (F(1, 9) = 32,275, p < 0.001) with adjusted R^2^ of 1.000 (Table 4).

**Table 3:** Surface difference of the medial tibial plateau models

Participants	KL grade	Surface difference	
1	1	0.09	[-0.15, 0.34]
2	1	0.10	[-0.16, 0.35]
3	1	-0.15	[-0.44, 0.14]
4	2	0.18	[-0.04, 0.40]
5	2	0.00	[-0.29, 0.30]
6	2	0.15	[-0.19, 0.49]
7	3	0.24	[-0.09, 0.57]
8	3	-0.02	[-0.32, 0.27]
9	3	0.20	[-0.09, 0.48]
10	3	-0.05	[-0.33, 0.24]

Surface difference is shown as means [95% confidence interval]. KL grade: Kellgren- Lawrence grade

**Table 4:** Models of linears regression analyses and results of each analysis

Model			Unstandardized coefficients		Standardized coefficients	t	Significant level
Outcome	Independent variable	Dependent variable	B	Standard error	Beta		
Volume of the medial tibial plateau	CT-derived model	MRI-derived model	0.978	0.011	0.999	89.527	0.000
Cross-sectional area of the medial tibial plateau	CT-derived model	MRI-derived model	1.007	0.006	1.000	179.653	0.000
MME volume	CT-derived model	MRI-derived model	0.983	0.200	0.998	49.932	0.000
MME width	CT-derived model	MRI-derived model	1.063	0.023	0.998	46.107	0.000
MME width	3D model	2D MRI slice	0.963	0.134	0.923	7.183	0.000

MME: medial meniscal extrusion, 3D: three-dimensional, and 2D: two-dimensional

The MME volumes of the CT- and MRI-derived models were 942.6 [597.7, 1287.6] mm^3^ and 916.2 [557.9, 1274.6] mm^3^, respectively (*p* = 0.938, d = 0.05, power = 0.91) (Table 2). The MME widths of the CT- and MRI-derived tibial models were 4.2 [1.9, 6.5] mm and 4.5 [2.2, 6.9] mm, respectively (*p* = 0.967, d = 0.12, power = 0.80). MME width measured on a 2D MRI slice was 5.5 [4.3, 6.7] mm. In addition, linear regression analysis demonstrated that the MME volumes on the CT- and MRI-derived models showed a high consistency (F(1, 9) = 2493, *p* < 0.001) with adjusted R^2^ of 0.996 (Table 4). Linear regression analysis also demonstrated that the MME width of the CT- and MRI-derived models showed a high consistency (F(1, 9) = 2126, p < 0.001) with adjusted R^2^ of 0.995. Lastly, linear regression analysis demonstrated that MME width on the 2D MRI slices and 3D models had an excellent consistency (F(1, 9) = 51.6, p < 0.001) with adjusted R^2^ of 0.835.

## Discussion

The most important findings in this study were that surface differences of the medial tibial plateau ranged from − 0.15 [− 0.44, 0.14] to 0.24 [− 0.09, 0.57], and that there was no significant difference between the MME volume calculated using the CT- and MRI-derived tibial models, and no significant difference between the MME widths calculated using the CT- and MRI-derived tibia. The consistency of the MME volume and the MME was excellent on linear regression analysis. 2D MME width was 5.5 [4.3, 6.7] mm, and the coefficient of determination adjusted R^2^ was lower than that of the MME width on CT- and MRI-derived models.

Accurate MME measurement requires accurate contours of the tibial plateau with the longitudinal osteophytes taken into consideration. Neubert et al. [17] showed that surface differences between CT- and MRI-derived models of the entire tibia ranged from 0.45 ± 0.38 mm to 0.83 ± 0.55 mm. The surface of the CT-derived femur of sheep were 0.23 mm greater than MRI-derived models [8]. Moro-oka et al. [18] reported that mean surface differences between CT- and MRI-derived models of the femur and tibia were 0.08 mm and 0.14 mm, respectively. The average [95% confidence interval] surface difference of the medial tibial plateau in this study was 0.24 [− 0.09, 0.57] in the subject showing the greatest difference. Therefore, surface differences between CT- and MRI-derived models in this study were similar to those found by Rathnayaka et al. [8]. In addition, there was no significant difference in cross-sectional area of the medial tibial plateau between CT- and MRI-derived models. Therefore, the MRI-derived medial tibial plateau model is as accurate as the CT-derived model for measurement of the MME and a difference in the MME width greater than 0.57 mm would be considered reliable.

Longitudinal comparison of the MME requires reasonable management of osteophyte growth over time. Hayeri et al. [19] observed osteophyte formation in the medial and lateral tibial compartments; 16 of 35 knees demonstrated osteophytes around the anterior part of the medial tibial plateau, while 12 of 35 showed osteophytes along the posterior part. Nagaosa et al. [20] observed osteophyte formation with attention to the orientation and found that osteophytes on the medial tibial plateau were oriented medially (73.1%), infero-medially (14.3%), or supero-medially (8.4%). Therefore, regions and directions of osteophytes on the medial plateau may vary across patients with KOA. In addition, Zhu et al. [21] showed that MRI detected osteophytes in 85% of participants, while X-ray image detected only 10% at the baseline. Using T2 mapping, Hada et al. [11] showed that osteophyte width including the cartilage corresponded to the actual measurement of the osteophyte tissue obtained during total knee arthroscopic surgery. Although these difference in detecting osteophytes may affect longitudinal comparisons of the MME, there has not yet been any longitudinal study investigating the effects of osteophyte growth on the accuracy of MME measurement over time. Therefore, the reference point on the tibial side for the measurement of the MME must be a point without osteophytes in order to minimize any bias caused by osteophyte growth. This study selected the reference point at the level of 10.0 mm. By selecting the plane without the effects of osteophyte, this technique can measure the MME more correctly especially in a longitudinal study. Moreover, this technique may allow to measure the morphological changes of the osteophytes. Therefore, the natural course of the biological adaptation can be measured. In the case of patients with greater osteophyte over this reference point, this method would not be adopted.

There has not been a previous study that compared MME measurements obtained from CT- and MRI-derived tibial bone models combined with an MRI-derived MM model. In patients with medial KOA, MME width on 2D MRI measured 4.3 ± 2.5 mm [16]. MME width in patients with KL grade 2 or 3 was reportedly 2.64 ± 1.10 mm when a method of excluding osteophytes was used during the segmentation process [13]. However, the method of identifying the osteophyte contours was not described in detail and there may have been a potential bias in detecting the tibial contour. In the non-weight-bearing knee of patients with KOA, ultrasonographic measurement of the MME was 6.12 ± 2.57 mm [2]. These researchers tried to exclude osteophytes by connecting the medial contour of the cortical bone of the tibia and femur [2]. This method may produce an error on longitudinal measurements of the MME due not only to the 2D method used but also to potential longitudinal changes in the femorotibial lateral translation [22] or lateral translation and adduction [23, 24] of the tibia on the coronal plane. This study measured the MME using a reference point on the contour of the tibial condyle 10 mm below the tibial plateau plane where there was less possibility to influence osteophytes. Since the selected contour was considered unlikely to be affected by the longitudinal growth of the osteophytes, measurement method in this study may be more accurate in measuring the MM displacement from the original meniscal position, not relative to the edge of the osteophyte, but from the contour of the young tibial plateau without osteophytes.

In this study, there was no significant difference in MME measurements between the CT- and MRI-derived tibial bone models. However, the average surface difference of 0.24 [− 0.09, 0.57] mm at maximum would likely be a source of systematic error and should be taken into consideration when comparing MME measurements between the CT- and MRI-derived tibial bone models. From the above, the MRI-derived tibial models provided reasonably accurate measurements of the MME volume and width after removing the influence of tibial plateau osteophytes. The internal validity of this study was high, and the methods used in this study are available for MME measurement in the early-to-moderate stages of primary medial KOA.

This study has a few limitations. First, the MRI sequences of this study were not specialized for analyzing bone morphology. This study employed a clinical sequence for analyzing medial meniscal disorder. Surface differences were similar to the findings of previous studies. Secondly, this study employed coronal slices in the MRI and the slice pitch was 2.0 mm. This may have affected the anterior or posterior margin of the MM, which can cause some error in volume and lesser extent in distance. Therefore, caution is necessary when comparing data from different studies due to errors caused by the segmentation method on MRI and when comparing 2D and 3D measurements of the MME. Thirdly, manual segmentation of the MM was done by single researcher. One observer segmentation has an advantage in reducing inter-observer errors, which is desired in a small study with 10 samples. However, it has a limited generalizability. Fourthly, this study applied the level of 10.0 mm as the reference cut point of the tibial plateau model. All the tibia in this study had no visual osteophyte at the level. However, careful attention would be needed when analyzing the MME with greater osteophyte in the tibial plateau.

## Conclusions

In conclusion, this study showed that the medial tibial plateau does not demonstrate significant morphological difference between the CT- and MRI-derived models, and that both models can be used as a reference to measure the MME in early-to-moderate medial KOA. This study showed the validity of the MME measurement method using the MRI-derived 3D tibial models after excluding the influence of tibial plateau osteophytes. Further studies are required to determine longitudinal changes in MME after eliminating the effects of osteophyte growth. This study concluded that the morphology of the medial tibial plateau does not demonstrate significant differences between CT- and the MRI-derived models, and that both the CT- and MRI-derived tibial model can be used as a reference when measuring MME in early-to-moderate medial KOA.

## Data Availability

The datasets used and/or analyzed during the current study are available from the corresponding author on reasonable request.

## Abbreviations

2D: Two-dimensional
3D: Three-dimensional
CT: Computed tomography
ICC: Intraclass correlation coefficient
KL: Kellgren-Lawrence
KOA: Knee osteoarthritis
MM: Medial meniscus
MME: Medial meniscal extrusion
MRI: Magnetic resonance imaging
RCT: Randomized controlled trial
